# Genetic Switch Underlies *C. albicans* Quick Change Act

**DOI:** 10.1371/journal.pbio.1001108

**Published:** 2011-07-19

**Authors:** Erik Vance

**Affiliations:** Freelance Science Writer, Mexico City, Mexico

**Figure pbio-1001108-g001:**
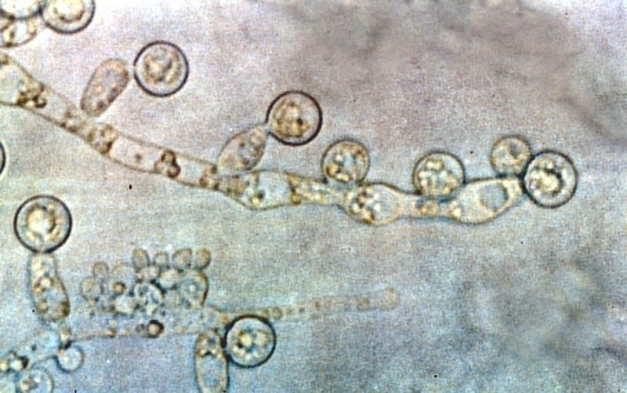
New research supports a new model to explain how *C. albicans*, the most common cause of invasive fungal infections in humans, manages the morphological plasticity necessary for pathogenesis.

[Fig pbio-1001108-g001]In the fungus world, just as in the business one, the key to success is flexibility. A fungus that can adapt and change to its environment is positioned to leap into new opportunities ahead of the competition.

If this is true, then *Candida albicans* is the Starbucks of fungi. *C. albicans* is a small, mobile fungus present in all of us, usually in the gastrointestinal tract or the mucus membrane. Most fungi exist in one of two forms—hypha or yeast. However, unlike most fungi, *C. albicans* can freely switch between hypha and yeast in a matter of hours, triggered by environmental changes like temperature or cell density. In the yeast form, it is not terribly dangerous but is highly mobile, hitching a ride in blood flows or other body processes. The hypha form spreads faster, given its filamentous-like nature, but cannot, for example, move into the bloodstream, and its ill-effects are limited. This phenomenon, called dimorphism, allows the fungus to quickly take advantage of changes and quickly adapt before its neighbors. Scientists have long struggled to understand the mechanisms that allow *C. albicans* to quickly slip between hypha and yeast phase. This week in *PLoS Biology*, a team of researchers from the University of California Irvine led by Haoping Liu provide a crucial new model for understanding the phenomenon.

In most people, *C. albicans* is just another member of the body's flora, harmlessly coexisting with other microorganisms in their host. Bacteria in particular are important competitors that keep its spread in check. However, if the environment suddenly changes, causing bacteria to die off, *C. albicans* quickly adapts and takes over. Usually the effects are minor. Elderly people who use dentures may experience a dry or burning mouth condition known as “thrush.” Women who suffer from irritating yeast infections and most babies fussing over diaper rash are the victims of an overaggressive population of *C. albicans*. However, if the patient's white blood cells have been compromised, say because of chemotherapy or an aggressive steroid treatment, the effects can be devastating. Serious complications arise if the fungus enters the bloodstream, perhaps through a catheter, which *C. albicans* can only do while in the more benign yeast form. However, once in the bloodstream, the fungus can deposit throughout the body as invasive hyphae. At that point it becomes known as “hematogenously disseminated candidiasis,” which proves fatal in 40% of cases, even with the best modern treatments.

The key to *C. albicans*' success is that it is highly adapted to change, switching between forms several times while its microbial neighbors must remain either one or the other. Sometimes the fungi will even switch as self-defense, using hyphal structures to fend off attackers. As a result, scientists have long tried to understand the mechanisms that allow *C. albicans* to quickly slip between hypha and yeast phase. A team at the University of California Irvine has painstakingly narrowed down the process to a series of two sequential genetic triggers that give this fungus its unique advantage.

First, they found that the primary obstacle to a swift yeast-hypha switch is a protein that inhibits transcription called Nrg1. The team found that before *C. albicans* transitions into the hyphal stage, it expunges Nrg1 by activating a major growth signaling pathway called cAMP-PKA.

After the Nrg1 was evacuated, the cell began to change its shape as well as its transcriptional program. But in order to prevent Nrg1 from returning to its position on the DNA—and keep the transition process moving without interruption—another protein, called Hda1, was recruited to maintain transcription and prevent the Nrg1 from binding to DNA. Hda1 achieved this by modifying, or deacetylating, the histone proteins that wrap around the DNA.

The authors demonstrate this model by creating mutant strains of *C. albicans* that could not produce Hda1 and thus were unable to complete the transition to the hyphal stage, despite the conditions being otherwise perfect (many things can induce a change, but the team used the most reliable—raising the temperature to 37°C and mixing in blood serum). The team also found that for a successful transition to hyphae, the yeast required rich, nutrient-packed media and that in nutrient-poor media, the hyphae simply stayed hyphae.

Liu and her team have created a new framework to look at dimorphism. Their results shed light on a mysterious process that is a major factor in lethal hospital infections. It also may aid in finding new treatments for diseases related to *C. albicans* if scientists could find a way to trap the fungus in its yeast form. It's also possible, now that scientists understand how the mechanism works, that they'll find out that many other animals use similar tools to perform a variety of tasks.

**Lu Y, Su C, Wang A, Liu H (2011) Hyphal Development in *Candida albicans* Requires Two Temporally Linked Changes in Promoter Chromatin for Initiation and Maintenance. doi:10.1371/journal.pbio.1001105**

